# EFFECT OF AN AI-BASED NEGOTIATION TRAINING FOR FAMILY CAREGIVERS OF OLDER ADULTS WITH ALZHEIMER’S DISEASE

**DOI:** 10.1093/geroni/igae098.3128

**Published:** 2024-12-31

**Authors:** Alaine Murawski, Celie Joblin, Marianne Tschoe, Vanessa Ramirez-Zohfeld, Charlie Olvera, Johnathan Mell, Jeanne Brett, Lee Lindquist

**Affiliations:** Northwestern University, Evanston, Illinois, United States; Northwestern University, Chicago, Illinois, United States; Northwestern University, Evanston, Illinois, United States; Northwestern University, Evanston, Illinois, United States; Northwestern University Feinberg School of Medicine, Division of Geriatrics, Evanston, Illinois, United States; Custom Technologies LLC, Orlando, Florida, United States; Northwestern University, Evanston, Illinois, United States; Northwestern University, Evanston, Illinois, United States

## Abstract

Family caregivers (CGs) of older adults with ADRD (OADRD) may experience conflicts when providing care, yet are often ill-equipped to handle these challenges. We evaluated NegotiAge (NA), a web-based intervention leveraging artificial intelligence (AI) to teach CGs negotiation skills, and assessed the impact that NA had on how CGs resolve conflicts and retain negotiation knowledge. CGs of OADRD were recruited nationally and enrolled in a randomized controlled trial. CGs accessed negotiation training resources through NA and were randomized to negotiate conflict scenarios in real-time, with AI-powered avatars exhibiting emotions and behaviors simulating real-life interactions. CGs were assessed at baseline (T1) and 1-week post-intervention (T2) with the Dutch Test for Conflict Handling (which examines yielding, compromising, forcing, problem-solving, and avoiding conflict domains) and with a 15-item panel consisting of negotiation terminology and conflict statement (interests, rights, power) identification. Matched pair t-tests were performed between T1-T2. 119 CGs (mean age 53.5, 75.6% female, 32.8% Black) completed both surveys. CGs receiving more negotiation exercises scored lower in avoiding (Δ=-0.97, t=-2.2, p=0.03) and compromising (Δ=-0.53, t=-3.5, p=0.0006) at T2. Mean total negotiation knowledge score increased between T1-T2 (Δ= 2.07, t=1.18, p = 0.07), as did identification of interests-based conflict statements (Δ=4.49, t= 4.48, p = 0.01). NA positively impacted CG conflict resolution skills: CGs improved both in avoiding and compromising during conflict, and in identifying common interests, essential to determining root causes of conflicts. Future research will examine NA’s impact on long-term CG outcomes (e.g. well-being, stress, burden) and care of the OADRD.

